# Rare Earth Ion-Doped Upconversion Nanocrystals: Synthesis and Surface Modification

**DOI:** 10.3390/nano5010001

**Published:** 2014-12-25

**Authors:** Hongjin Chang, Juan Xie, Baozhou Zhao, Botong Liu, Shuilin Xu, Na Ren, Xiaoji Xie, Ling Huang, Wei Huang

**Affiliations:** 1 Key Laboratory of Flexible Electronics (KLOFE) and Institute of Advanced Materials (IAM), Jiangsu National Synergistic Innovation Center for Advanced Materials (SICAM), Nanjing Tech University (NanjingTech), Nanjing 211816, China; E-Mails: 460653533@njtech.edu.cn (H.C.); 823184209@njtech.edu.cn (B.Z.); liubotong201304@gmail.com (B.L.); xu_shuilin@njtech.edu.cn (S.X.); iamnren@njtech.edu.cn (N.R.); iamxjxie@njtech.edu.cn (X.X.); 2 Key Laboratory for Organic Electronics and Information Displays and Institute of Advanced Materials (IAM), National Synergistic Innovation Center for Advanced Materials (SICAM), Nanjing University of Posts and Telecommunications, Nanjing 210023, China; E-Mail: 15050528303@163.com

**Keywords:** rare earth, nanocrystal, upconversion, synthesis, surface modification

## Abstract

The unique luminescent properties exhibited by rare earth ion-doped upconversion nanocrystals (UCNPs), such as long lifetime, narrow emission line, high color purity, and high resistance to photobleaching, have made them widely used in many areas, including but not limited to high-resolution displays, new-generation information technology, optical communication, bioimaging, and therapy. However, the inherent upconversion luminescent properties of UCNPs are influenced by various parameters, including the size, shape, crystal structure, and chemical composition of the UCNPs, and even the chosen synthesis process and the surfactant molecules used. This review will provide a complete summary on the synthesis methods and the surface modification strategies of UCNPs reported so far. Firstly, we summarize the synthesis methodologies developed in the past decades, such as thermal decomposition, thermal coprecipitation, hydro/solvothermal, sol-gel, combustion, and microwave synthesis. In the second part, five main streams of surface modification strategies for converting hydrophobic UCNPs into hydrophilic ones are elaborated. Finally, we consider the likely directions of the future development and challenges of the synthesis and surface modification, such as the large-scale production and actual applications, stability, and so on, of the UCNPs.

## 1. Introduction

On 29 December 1959, Richard Feynman predicted at the annual American Physical Society meeting that: “*if we use a method to control the arrangement of things at a small scale, so that we can get a lot of features beyond imagination, also can see the performance of the material to produce rich change.*” The tiny material is what we call nanomaterial today, which is usually referred to as that in the three dimensional space, where at least one dimension (1D) is at the nanoscale range (1–100 nm) or materials are composed of them as a basic unit. As the size of the material decreases to the nanometer scale, the material will show a lot of new features that the bulk counterpart does not possess, such as the small size effect, surface effect of nanomaterials, quantum confinement effect, and macroscopic quantum tunneling effect [1,2,3]. In the past decades, nanomaterials have gained wide attention from all over the world, and have now been widely applied in many fields.

Rare earth (RE) elements possess unique electronic configuration where the 4f electrons are effectively shielded by the closely lied 5s and 5p subshells. Typically, the electron transitions of RE ions are mainly derived from the inner *4f*-*4f* or *4f*-*5d* transitions, and thus the spectroscopic properties of RE ions are barely perturbed by the local chemical microenvironment, which imparts RE compounds including the complexes’ and nanomaterials’ unique spectroscopic characteristics, such as rich energy levels, long luminescence decay time, narrow emission line, and high color purity in contrast to those of quantum dots and organic dyes [4,5,6]. Due to the preserved electron transitions, the emission wavelengths of each lanthanide ion largely depend on its own electronic configuration, and the combination (at certain ratio) of different lanthanide ions is also widely adopted to realize various luminescent materials with adjustable luminescence for different purposes.

If these RE ions were doped into proper nanocrystals, a series of new luminescent characteristics related with the original features of according RE ions may be observed. In addition, upconversion nanocrystals (UCNPs) have shown high chemical stability, biological compatibility, long luminescence lifetime, and tunable emission wavelength [7,8,9], making them tremendously exploited in biolabeling [10], bio-detection [11,12], bioimaging [13,14], FRET-based sensing [15], drug delivery [16], and volumetric 3D display [17].

Despite the substantially shielded transitions of RE ions, the luminescent properties of UCNPs are also affected by their size, shape, crystal structure, and chemical composition of the materials. For example, reduced particle size will cause increased surface area, which would introduce more defects on the nanoparticle surface and consequently hamper the luminescent efficiencies, though the small nanoparticles are more advantageous to the biological applications. To facile the applications of UCNPs, it is of primary necessity to develop according viable and robust methodologies to synthesize target nanocrystals with desired size, shape, crystal structure, chemical composition, and most importantly, the proper surface functional groups for anticipated applications.

In recent years, various attempts have been reported for synthesizing UCNPs in a controlled manner, including thermal decomposition, thermal coprecipitation, hydro/solvothermal, combustion, microwave, and so on. In the meantime, the nanocrystals, after surface modification, such as SiO_2_ encapsulation, polymer encapsulation, ligand oxidation, and ligand exchange, can be easily coupled with DNA, protein, and other functional molecules, and facilitate expected applications. However, there are still some challenges in the synthesis of desired nanocrystals, and the surface modification usually involves extra experimental steps and lowers the luminescence efficiency at certain extent depending on the chosen method. Thus, a proper synthesis method and a suitable strategy for designed surface modification are highly desired. Herein, we attempt to provide a comprehensive overview of the state-of-the-art synthetic methods and the surface modification strategies for UCNPs reported in the past decades.

## 2. Synthetic Approaches

### 2.1. Thermal Decomposition

**Figure 1:**
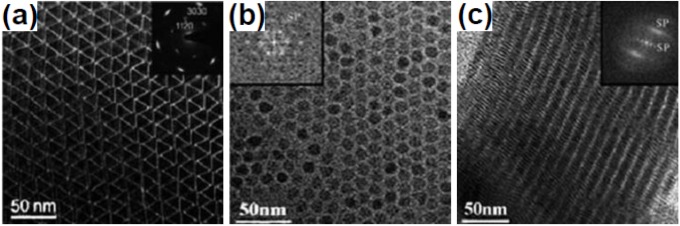
(**a**) TEM image of edge-to-edge super lattices of LaF_3_ triangular nanoplates. Reproduced from [18]. Copyright 2005, American Chemical Society. (**b,c**) TEM images of edge-to-edge and face-to-face super lattices of SmF_3_ hexagonal nanoplates, respectively. Reproduced from [19]. Copyright 2006, John Wiley and Sons.

The thermal decomposition process, comprising of dissolution of organic and/or inorganic precursors in organic solvent with high-boiling point, is a traditional method for preparation of inorganic nanocrystals. The typical experimental procedure of thermal decomposition method is composed of: (1) a given amount of RE(CF_3_COO)_3_ precursors is added into a mixture of oleic acid (OA), 1-octadecene (OD), and sometimes oleylamine (OM) at room temperature; (2) the solution is heated to 165 °C for 30 min with vigorous magnetic stirring to remove water and oxygen under argon protection; (3) the solution is heated to high temperature (usually >300 °C) for a certain period of time under argon protection and the nanocrytals are then collected from the reaction mixture after cooling down to room temperature. Yan [18,19] first prepared high-monodisperse LaF_3_ triangular nanoplates (Figure 1a) and hexagonal SmF_3_ nanoparticles (Figures 1b,c) via the thermal decomposition process using La(CF_3_COO)_3_ and Sm(CF_3_COO)_3_ as precursors. Using this method, Capobianco [20] and Nann [21] synthesized NaYF_4_ nanocrystals with narrow size-distribution. Later, this approach was extended as a common process to synthesize high-quality UCNPs including but not limited to NaLaF_4_ [22], NaGdF_4_ [23], LiYF_4_ [24], KY_3_F_10_ [25], BaYF_5_ [26], REOF [19,27,28], and REOCl [29]. The reaction temperature, time, and the molar ratio of OA, OD, and sometimes OM in the reaction mixture have been demonstrated to exert different effects on the final nanocrystals. It should be noted that sometimes OM shall be introduced as a necessary component to adjust the reaction environment so that the final product with different morphologies and dimensions or a brand new product can be obtained [27,28,29].

The most prominent advantages of this method are that the products are of high quality, with pure crystal phase, and strong upconversion emission. However, this method also suffers from disadvantages including: (1) presynthesis of RE(CF_3_COO)_3_ precursors is typically required; (2) the decomposition of trifluoroacetates simultaneously produces toxic fluorinated and oxyfluorinated carbon species and thus careful handling of the reactions in fully ventilated chemical hood is required; (3) the anaerobic and water-free reaction environment further increases the operation difficulty. In the themolysis method, one of the key factors for achieving size-tunable and monodispersed UCNPs requires a proper selection of the coordinating ligands. Yan and co-worker reported that oleylamine ligand is a delicate buffer for F^−^ ions, the lighter the rare earth, the more OM it requires, owing to the fact that the basicity of the RE oxide gradually decreases along with the increasing of the atomic number of the RE series [19].

### 2.2. Thermal Coprecipitation

Due to the limitations of the thermal decomposition method, thermal coprecipitation approach is developed and has now been used as one of the most convenient methods for UCNPs synthesis. The experimental procedure is generally composed of: (1) RE salts were mixed with a solution of OA, OD, and sometimes OM, at certain ratio, which was heated to 165 °C for 30 min and then cooled down to room temperature; (2) a methanol solution of NH_4_F and AOH (A = Li, Na, K) was added to the mixture and stirred for 30 min; (3) after removal of the methanol and residual water by evaporation, the reaction mixture was heated to high temperature (usually >300 °C ) under argon protection, which produces desired nanocrystals. The benefits of the coprecipitation method include operational simplicity, the lack of toxic by-products, and the wide application across various materials.

**Figure 2:**
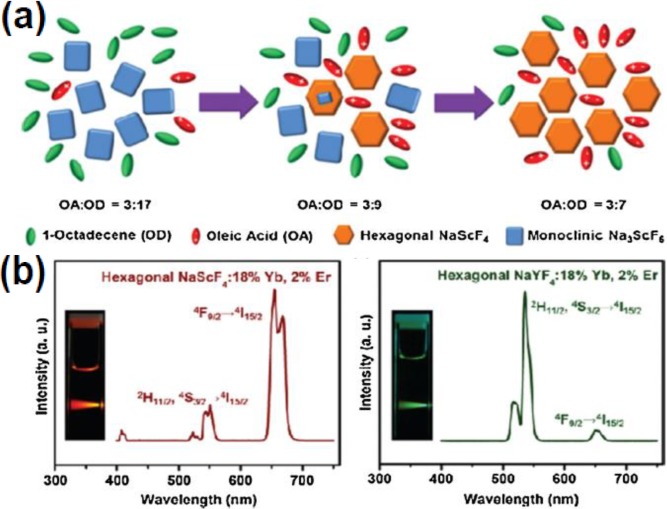
(**a**) Schematic illustration of the crystal structure evolution at varying polarities of the reaction medium. (**b**) Upconversion luminescence spectra of hexagonal-phase NaScF_4_:Yb/Er and NaYF_4_:Yb/Er nanocrystals. Reproduced from [35]. Copyright 2012, American Chemical Society.

With continuous efforts, many kinds of UCNPs have been directly synthesized by this method. For example, in 2002, van Veggel and co-workers [30] have synthesized the down conversion LaF_3_ nanocrystals doped with RE^3+^ (RE = Eu, Er, Nd, and Ho) ions. This approach was later expanded by Yi and Chow [31], who prepared upconversion LaF_3_ nanophosphors with smaller particle size (5 nm). In addition to LaF_3_, LuPO_4_:Yb/Tm, YbPO_4_:Er, NaYF_4_:Yb/Er(Tm), NaGdF_4_:Yb/Er, and Y_3_Al_15_O_12_(YAG):Yb/Tm nanophosphors were synthesized using this method [5,32,33,34]. Recently, our group has synthesized the first Scandium-based fluoride nanocrystals Na*_x_*ScF_3+*x*_:Ln using the coprecipitation method [35] (Figure 2). Interestingly, the crystal structure evolution from pure monoclinic Na_3_ScF_6_ to pure hexagonal NaScF_4_ phase was observed by tuning the ratio of OA and OD (Figure 2a). In addition, the hexagonal NaScF_4_:Yb/Er crystals emit strong red upconversion (665 nm) under 980 nm laser excitation, different from those of the traditional hexagonal NaYF_4_:Yb/Er nanocrystals, which usually emit strong green (547 nm) upconversion luminescence (Figure 2b).

Despite the general usefulness and obvious improvement of the thermal coprecipitation method for UCNPs synthesis, it suffers from the long-time and continuous operation of the experimental process, which usually takes more than 5 h, including the removal of methanol solvent, water generated during the synthesis, and the controlled crystal growth at a certain high-temperature. Moreover, large scale synthesis of UCNPs using this method is still a great challenge.

### 2.3. Hydro/Solvothermal Synthesis

Hydro/solvothermal is a process of chemical reaction between negative ions and positive ions that usually precipitate from the solvent under high temperature and high pressure, generating nanoscale materials in the solvent after proper processing. This method has now become a widely-employed synthetic approach for UCNPs since it is easy to operate, does not require stringent operation of the experimental process, and moreover, the reaction temperature of hydro/solvothermal method is usually lower than those used for thermal decomposition and thermal co-precipitation synthesis for UCNPs.

**Figure 3:**
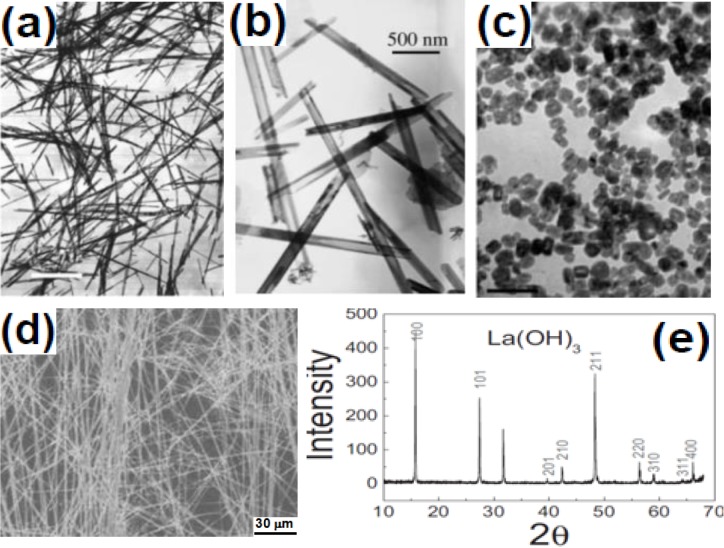
TEM images of (**a**) La(OH)_3_ nanowires. Reproduced from [47]. Copyright 2002, John Wiley and Sons; (**b**) Y_2_O_3_ nanotubes. Reproduced from [48]. Copyright 2003, John Wiley and Sons; (**c**) LaF_3_ nanoparticles. Reproduced from [49]. Copyright 2003, John Wiley and Sons; (**d**) La(OH)_3_ nanobelt; (**e**) a typical XRD pattern of the as-synthesized La(OH)_3_. Reproduced from [50]. Copyright 2007, John Wiley and Sons.

**Figure 4:**
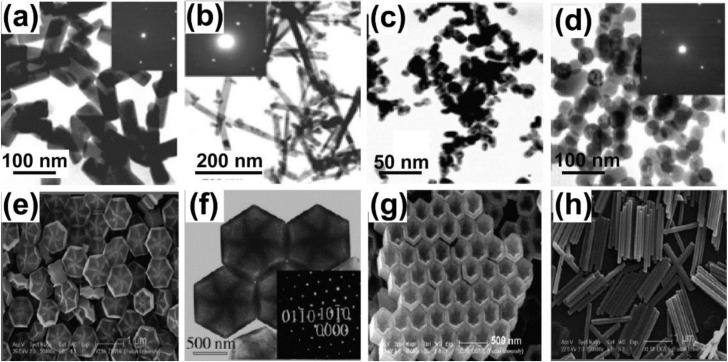
TEM and SEM images of NaYF_4_:Yb/Er nanocrystals prepared under different hydro/solvothermal conditions. (**a,b**) TEM images of NaYF_4_:Yb/Er nanocrystals synthesized in acetic acid and ethanol in the presence of CTAB, respectively; (**c,d**) TEM images of NaYF_4_:Yb/Er nanocrystals using EDTA in acetic acid and ethanol, respectively. Reproduced from [34]. Copyright 2005, John Wiley and Sons; SEM images of (**e,f**) flower-patterned hexagonal disks; (**g**) hexagonal nanotubes; and (**h**) nanorods of β-NaYF_4_. Reproduced from [51]. Copyright 2007, John Wiley and Sons.

The commonly used surfactants for UCNPs preparation include ethylenediamine tetraacetic acid (EDTA) [36,37], cetyltrimethylammonuim bromide (CTAB) [38], trisodium citrate (Na_3_Cit) [39], linoleate acid [40], oleic acid [41,42,43,44,45,46]. As early as 2002, Li and coworkers [47,48,49] used RE nitrate and KOH to synthesize Ln(OH)_3_ nanowires, nanotubes, and nanoparticles (Figure 3a–c). Long and uniform nanobelts were obtained when NaOH/KOH and RE acetates were used (Figures 3d,e). Later on, EDTA was used as complexing agent and CTAB as structure-directing agent to synthesize β-NaYF_4_:Yb/Er nanotubes, nanorods, and nanospheres [34] (Figure 4a–d). Different from Li’s work, Wang and co-workers [50] synthesized upconversion NaYF_4_ nanocrystals using oleic acid-mediated hydrothermal synthesis (Figure 4e–h).

Recently, via a hybrid thermal decomposition/solvothermal method, LaF_3_:Yb/Er/Tm/Ho nanoplates with multicolor upconversion luminescence were synthesized [51,52]. The feasibility of the hydro/solvothermal methods was demonstrated by several groups. For example, Zhang has synthesized BaYF_4_ [53], CePO_4_ [54], YPO_4_ [55], Gd_2_O_3_ [56], GdVO_4_ [57], and Lin’s group reported the uniform microstructured YPO_4_ [58], YVO_4_ [59], LnF_3_ [60], and NaYF_4_ [45] via the sodium citrate assisted hydrothermal route. In recent years, Liu and co-workers reported the synthesis of KMnF_3_ nanocrystals with only single-band UC emission [61]. Zhao and Hao’s group [62,63] represented a strategy for the rationale manipulation of green and red upconversion emission, and the pure red emission of NaYF_4_:Yb/Er nanocrystals has been achieved by Mn^2+^ doping.

However, the disadvantages of this method are substantially difficult to overcome. Firstly of all, there are too many parameters including the reaction temperature, surfactant type and concentration, reactant concentration, solvent and the composition of it, and reaction time, to consider in order to filter out the most optimized experimental conditions for one specific reaction. Furthermore, the obtained nanocrystals usually have large size distribution, and sometimes the by-product residues stay on the surface of the nanocrystals, which are difficult to remove. Therefore, it remains challenging to develop a general and facile hydro/solvothermal method for the synthesis of high-quality UCNPs.

### 2.4. Sol-Gel Method

Sol-gel process is a typical wet-chemical technique for synthesizing UCNPs, which can be generally divided into three types: (1) sol-gel route based on the hydrolysis and condensation of molecular precursors; (2) gelation route based on condensation of the aqueous solutions containing metal-chelates; and (3) polymerizable complex route [64]. In sol-gel process, a RE nitrate salt or metal alkoxide is generally used as the starting reactants. The reaction is started by mixing the reactants in liquid phase through hydrolysis and condensation reactions, followed by annealing at high temperature for a certain period of time.

**Figure 5:**
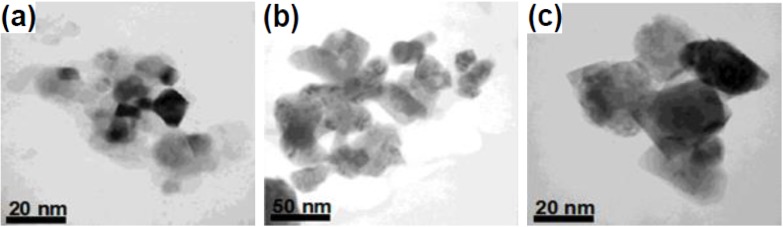
TEM images of the 1.0 mol% Er^3+^-doped BaTiO_3_ nanoparticles obtained after heating to three different temperatures: (**a**) 700 °C; (**b**) 850 °C; and (**c**) 1000 °C. Reproduced from [71]. Copyright 2003, American Chemical Society.

In 2002, Prasad *et al.* [65] developed a sol emulsion-gel method that produces Er^3+^ doped ZrO_2_ nanophosphors. In order to reduce the segregation of particles and ensure compositional homogeneity, Lin and co-workers fabricated an inorganic YVO_4_:Eu thin film phosphor by combining the pechini-type sol-gel process with inkjet printing. The mixed solution of metal salt precursors, citric acid, and poly(ethylene glycol) was directly used as ink to deposit patterns on ITO-coated glass substrate. After calcination at 600 °C in air, the YVO_4_:Eu patterns at the micrometer-scale were formed on the substrate [66]. In another report, Song and co-works successfully fabricated many kinds of inverse opal photonic crystals (PCs) by the sol-gel method with a PMMA latex sphere template, including YVO_4_:Dy [67], TiO_2_:Sm [68], YBO_3_:Eu [69], and LaPO_4_:Ce,Tb [70]. In addition, the sol-gel process was also developed for synthesizing various UCNPs using metal oxides as host materials such as TiO_2_:Er, BaTiO_3_:Er (Figure 5), Lu_3_Ga_5_O_12_:Er, and YVO_4_:Yb/Er [71,72,73].

For the sol-gel method, the annealing procedure (temperature and time) is the key step in the preparation process, which can seriously determine the quality of the samples. It should be noted that, although the sol-gel method can be used for large-scale production and the products usually offer high luminescence intensity due to the high crystallinity formed at high annealing temperature, the sol-gel derived nanocrystals generally have broad particle size distribution, irregular morphology, and are insoluble in water, which compose the shortcomings of this method.

### 2.5. Microemulsion

**Figure 6:**
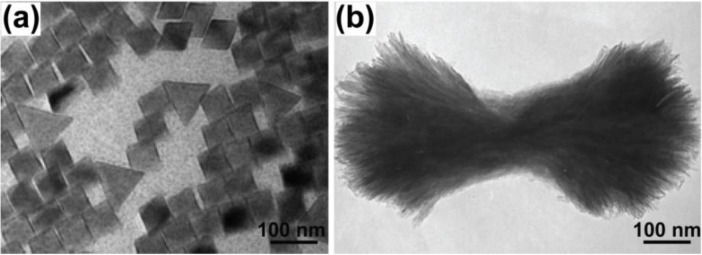
TEM images of (**a**) YF_3_ nanophosphors prepared in the NP-5 stabilized microemulsion system. Reproduced from [74]. Copyright 2005, American Chemical Society. (**b**) YF_3_ nanobundles synthesized in the CTAB and NP-5 stabilized microemulsion system. Reproduced from [75]. Copyright 2008, American Chemical Society.

To synthesize nanomaterials via the microemulsion method, it usually requires surfactant, co-surfactant, organic solvent, water, and the initial reagents. In a typical microemulsion solution, amphiphilic surfactants form a monolayer at the oil-water interface, with the hydrophobic tails of the surfactant molecules dissolved in the oil phase and the hydrophilic head groups in the aqueous phase. Preparation of RE fluoride nanomaterials usually needs two separate microemulsion systems and the two microemulsions containing RE ions and fluorine ions, respectively, get mixed to initiate the reaction. Over the past few years, Lemyre and coworkers [74] have reported that upconversion YF_3_ nanoparticles can be prepared in a reverse water-in-cyclohexane microemulsion system stabilized by polyoxyethylene isooctylphenyl ether (IGEPAL CO-520 or NP-5was supplied by Aldrich) (Figure 6a). Qin and coworkers [75,76] prepared YF_3_ upconversion nanophosphors, using CTAB and 1-pentanol instead of NP-5 (Figure 6b).

The use of microemulsion has many advantages, such as the low-cost for equipment, easy operation, the small size of the UCNPs, and the controlled morphology of products by adjusting the dosage of the surfactant, solvent, as well as the aging time. However, this technique has many challenges including the small amount of products generated, difficulty of sample separation and narrow scope synthesis. More importantly, it is difficult to achieve massive production to meet industry requirements.

### 2.6. Combustion Synthesis

Compared to sol-gel and hydro/solvothermal synthesis, combustion method for UCNPs synthesis can be finished in a short period of time. The process of combustion synthesis is an oxidation-reduction reaction in essence. Metal nitrates are usually selected as oxidizer and the source for metal ions while the organic compounds as reducing agent and fuel. There are two requirements for organic fuel selection: (1) the reaction occurred between the fuel and nitrate must be relatively mild, producing nontoxic gases; (2) it is better to select the fuels that can complex with the metal ions, enhance the solubility of metal ions, and prevent the separation by crystallization of the metal salts in the precursor solution.

During the combustion synthesis process, combustion wave spreads the reaction materials in a self-sufficient situation without requiring extra heat in the process of the whole reaction. This time- and energy-saving method was used to synthesize oxide and oxysulfide nanomaterials. For example, Capobianco, Luo, and Zhang’s groups have respectively synthesized a variety of oxide and oxysulfide nanophosphors (Y_2_O_3_, Gd_3_Ca_5_O_12_, La_2_O_2_S, and Gd_2_O_3_) [77,78,79,80] via this method.

### 2.7. Flaming Synthesis

Flaming synthesis is another powerful method for producing RE oxide nanomaterials, which can be divided into four stages: (1) precursor reaction; (2) nucleation; (3) growth and polymerization; and (4) ion deposition. Notably, the flaming synthesis differs from the typical combustion synthesis that all reactions take place in the gas-phase and form fine powders. The core advantages of the technique are time-saving and low-cost. In 2007, Ju [81] reported the synthesis of Y_2_O_3_:Yb/Er(Tm, Ho) nanophosphors using this method. However, since the oxidation reaction happens along with the flaming process, this method is mainly limited to oxide nanomaterial synthesis, and it is almost impossible to synthesize other types of UCNPs, such as fluoride, phosphate, vanadate, and so on. It should also be noted that the flaming synthesis offers an opportunity for large-scale synthesis of RE doped oxides.

### 2.8. Electrospinning

During the electrospinning process, the precursor solution is spun through four stages (cocoons, stretching, refinement, and curing) under the effect of high voltage electrostatic field. Simple operation, good repeatability, and wide application scope are the advantages of this approach. For example, Song [82] have reported NaYF_4_/PVP composite nanofibers, with diameter in the range of 300–800 nm, prepared through electrospinning. Nevertheless, this method is still in its infantile stage. New recipes for the synthesis of uniform and small-sized (<20 nm) nanoparticles with controllable morphology are still required.

### 2.9. Microwave Synthesis

Microwave synthesis contains solid microwave and liquid microwave method. The former mixes up RE oxides with ammonium bifluoride and ammonium fluoride, and the nanoparticles were directly synthesized via microwave. The latter dissolves the RE salts and the fluoride source into solvent. Then, these raw materials react with each other when the solvent is heated by microwave. The method was extended to produce PrF_3_ hemispheres with diameter of about 31 nm [83].

The advantage of microwave synthesis is generally composed of: (1) it can selectively heat the samples to high temperature while the rest of the microwave device remains at room temperature; (2) microwave can heat the reaction system uniformly, cause less side reaction and the product is relatively simple; (3) fast heat and low energy consumption; (4) improve the structure and properties of synthetic material by adding proper surfactant.

Besides the above synthetic strategies elaborated, combinatorial approaches have also been employed to produce micro- or nano-scale RE fluoride crystals, which after proper lanthanide doping, can upconvert near-infrared light to visible frequencies, enabling the applications of such materials to biological imaging, telecommunication, and solar energy conversion [84]. For example, Liu [85] have synthesized NaYF_4_:Yb,Er upconversion nanocrystals with characteristic upconversion luminescence spectra in a continuous capillary. Later, the research group improved the condition of the reaction prevented the growth of β-NaYF_4_ [86]. On the other side, Zhu *et al.* synthesized LaF_3_ [87] and LaPO_4_ [88] nanoparticles doped with Ce^3+^ and Tb^3+^ using microcapillary flow reactors heated with microwaves. However, they also suffer from the wide quality variation of the UCNPs synthesized from different vessels and the large-scale production.

## 3. Surface Modification of UCNPs

Because of the influence of impurities and lattice defects on the synthesized UCNPs, the quantum yield of UCNPs is lower than the corresponding bulk materials. In addition, the UCNPs are mostly insoluble in water since they are prepared from the organic environment and usually surrounded by hydrophobic surfactant molecules. Thus, it is important to develop appropriate strategies to make them hydrophilic and in the meantime maintain their upconversion efficiency to satisfy various purposes. For example, the ideal luminescent nanocrystals used for biocompatible purposes should meet several requirements, including: (1) high luminescence efficiency and low background noise; (2) good solubility and stability in biological environment; (3) good biological compatibility; and (4) proper size (below 100 nm).

However, a notorious weak point of UCNPs is the inherent low upconversion efficiency. Currently, the urgent task is to improve the upconversion efficiency, and there have been several groups working on this topic via the core-shell strategy.

The introduction of an inert crystalline shell of an undoped material around each doped nanocrystal provides an effective option to improve the luminescence efficiency of UCNPs. The shell usually has the same composition as the core host crystal, which can effectively reduce the surface quenching effect. In such structures, all dopant ions are confined in the interior core of the nanocrystals, effectively suppressing the non-radiative energy transfer from RE ions to the surface quenching sites, which results in improved upconversion luminescence efficiency. A significant demonstration was made by Yi and Chow [89] who reported a luminescence enhancement of nearly 30 times on 8 nm NaYF_4_:Yb/Tm nanocrystals coated with a 1.5 nm thick NaYF_4_ shell. Later on, Chen and co-works exploited a strategy to achieve dual-mode luminescence from identical Eu^3+^ ions in monodisperse hexagonal-phase NaGdF_4_ nanocrystals that consist of the NaGdF_4_:Yb/Tm core and the NaGdF_4_:Eu shell. Typical red downconversion luminescence of Eu^3+^ has been detected via the sensitization of Gd^3+^ ions. By using Yb^3+^ and Tm^3+^ embedded in the cores as double sensitizers, intense upconversion luminescence of Eu^3+^ in the shells can be achieved in NaGdF_4_:Yb/Tm@NaGdF_4_:Eu core-shell nanocrystals upon excitation at 976 nm. The upconversion intensity of Eu^3+^ in core-shell nanocrystals is found about one order of magnitude higher than the counterparts of the triply-doped core only, due to the inhibition of the deleterious cross-relaxations between Tm^3+^ and Eu^3+^ ions in core-shell nanocrystals that are reasonably separated in space [90].

**Figure 7:**
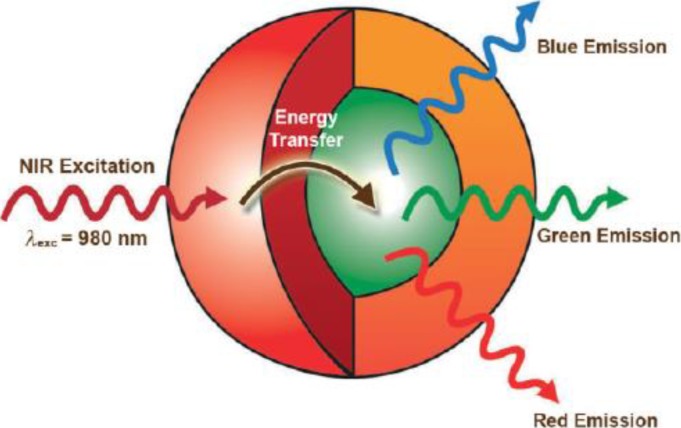
Schematic illustration of the active core-active shell nanoparticle architecture showing the absorption of NIR near infrared light by the Yb^3+^-rich shell (red) and subsequent energy transfer to the Er^3+^,Yb^3+^-doped core (green), which leads to upconverted blue, green, and red emissions. Reproduced from [91]. Copyright 2009, John Wiley and Sons.

Recently, Capobianco [91] proposed a strategy to significantly enhance the intensity of the upconversion by employing a novel NaGdF_4_:Yb/Er@NaGdF_4_:Yb active-core@active-shell architecture (Figure 7). The active-shell serves two purposes: (i) minimize the quenching centers and channels; and (ii) transfer absorbed near infrared energy to the luminescence centers (emitters).

Utilizing the core-shell strategy, Zhang and co-workers [92] have broken through the well accepted upper limit of the concentration quenching threshold, e.g., from ~2 mol% to 5 mol% for Er^3+^, by a designed multi-layer core-shell structure, which contains four parts: the illuminating core (NaYF_4_:Yb/Er), the first separating shell (NaYF_4_:Yb), the second illuminating shell (NaYF_4_:Yb/Er) and the final inactive shell (NaYF_4_:Yb). The separating layer effectively inhibits the energy transfer process between the Er^3+^ ions in the inside and outside layers and largely reduces the possibility of excitation energy trapping by defects, resulting in an effective upconversion luminescence enhancement [92]. Other core-shell examples include CeF_3_:Tb@LaF_3_ [93], NaYF_4_:Yb/Er@NaYF_4_ [89,94], NaGdF_4_:Yb/Er@NaGdF_4_ [95], and KYF_4_@KYF_4_:Yb/Er [96]. Liu [97] introduced a rationale core-shell strategy that provides precise control over the concentration of dopant in the core and shell layers of nanoparticles. A small amount of Nd^3+^ ions is doped into the core, while a high concentration of Nd^3+^ ions (~20 mol%) is selectively doped in the shell layers for effective harvesting of light at 800 nm. Chen and co-workers developed a facile strategy based on successive layer-by-layer (LBL) injection of shell precursors for the synthesis of LiLuF_4_:Ln^3+^ core-shell UCNPs [98]. It is reported that the size of nanocrystals can be tuned by adjusting the amount of NH_4_F (the more the amount of NH_4_F solution used, the bigger the size of crystals) and the shell thickness of core-shell nanocrystals can also be controlled by the volume of the core [99]. Besides, Yan has proved in the structure of NaYF_4_:Yb,Er@CaF_2_ that the thickness of the CaF_2_ shell were controlled by adjusting the [Ca]/[RE] ratio [100]. Although this approach provides tunable emission intensity in nanocrystals, the luminescence quantum yields of the nanocrystals are limited due to the weak ligand fields and high energy oscillations. Further improvement of the luminescence efficiency of UCNPs can be expected through the controlled growth of a rationally designed crystalline inner shell. Generally speaking, such core-shell structure improves the optical properties of the nanocrystals, but usually does not change the chemical functional group binding on the surface. So, proper surface modification is needed in order to make practical applications of UCNPs, especially for bio-related purposes.

### 3.1. SiO_2_ Encapsulation

**Figure 8:**
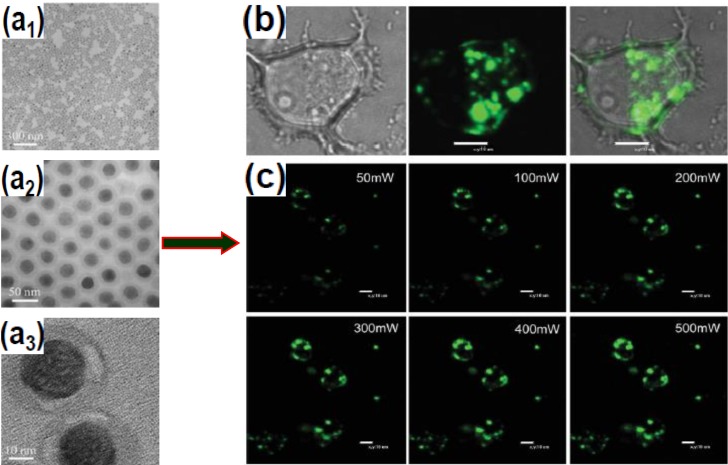
Silica-coated NaYF_4_:Yb/Er nanocrystals and their application for cell imaging. (**a**1–C**a**3) TEM images of silica-coated NaYF_4_:Yb/Er UCNPs upconversion nanoparticles at different magnifications; (**b**) Confocal fluorescence image of MCF-7 cells using silica-coated NaYF_4_:Yb/Er nanospheres (Left: bright-field, middle: upconversion image under 980 nm excitation, and Right: superimposed images of MCF-7 cells incubated with the nanoparticles for 24 h); (**c**) Confocal fluorescence images of MCF-7 cells with the nanospheres, excited by a 980 nm laser with different power intensities. Reproduced from [103]. Copyright 2008, John Wiley and Sons.

SiO_2_ encapsulation involves the growth of an amorphous silica shell on the UCNPs core. Due to the rich –OH groups on the SiO_2_ coated UCNPs, it becomes possible to further functionalize the UCNPs with expected chemical groups such as –NH_2_, –COOH, polymers, and more importantly a wide variety of biomolecules. Nann and Capobianco [101] obtained surface-functionalized YF_3_ nanocrystals using this technique. One year later, Zhang and coworkers [102] succeeded in growing a silica shell with adjustable thickness in the range of 2–10 nm on the surface of PVP stabilized cubic NaYF_4_:Yb/Er/Tm nanocrystals. Later on, they used the microemulsion method to coat NaYF_4_ nanocrystals with SiO_2_, which resulted in monodispersed SiO_2_-coated UCNPs (Figure 8) [103].

Alternatively, Song and co-workers [104] showed the conversion of hydrophobic NaYF_4_:Yb/Er upconversion nanophosphors into hydrophilic ones by amphiphilic silane modification with ultrathin thickness (~1 nm) at room temperature. In this strategy, the coating layers can also provide the possibility for loading Eu(TTA)_3_(TPPO)_2_ complex with downconversion luminescence where they realized the dual mode temperature sensing and dual mode cell imaging within the physiological environment [104]. There are other successful examples to modify the surface of the UCNPs [105,106,107,108] via the SiO_2_ encapsulation method, such as LaF_3_:Ln@SiO_2_, NaYF_4_:Yb/Er/Fe_3_O_4_@SiO_2_, NaYF_4_:Er,Tm,Ho@SiO_2_, NdF_3_@SiO_2_, and NaYF_4_@SiO_2_@ quantum dots [103].

The SiO_2_ coating provides a versatile platform for multi-functionalization of UCNPs. Nevertheless, the method is time consuming and difficult for large scale synthesis. On the other hand, the coated SiO_2_ layer may also affect the luminescence intensity of the UCNPs through light scattering, which prevents it from becoming the ideal surface modification recipe because of the already low upconversion efficiency of the UNCPs. Thus, there is still much work to do to improve the general applicability of this technique.

### 3.2. Polymer Encapsulation

Polymer encapsulation typically involves the absorption of an additional amphiphilic polymer onto the nanophosphor surface through the hydrophobic-hydrophobic attraction between the original surfactant molecules and the hydrocarbon chains of the polymer. Using this strategy, Chow and co-workers successfully coated a polyacrylicacid (PAA) layer on NaYF_4_:Yb/Er@NaYF_4_ core-shell nanoparticles [89]. The hydrophobic core-shell nanoparticles were rendered hydrophilic by amphiphilic PAA coating.

**Figure 9:**
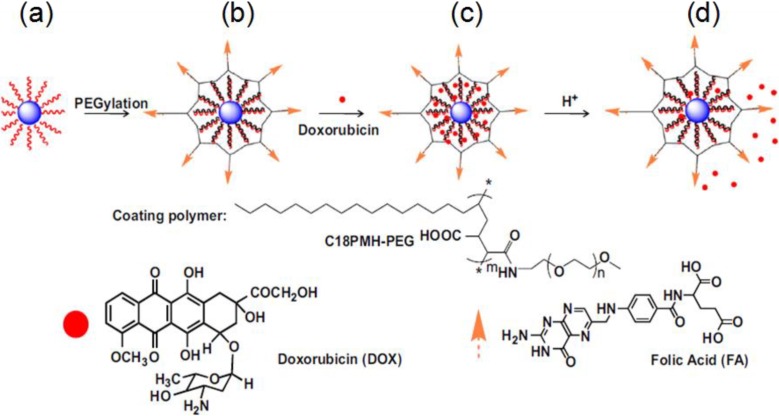
Schematic illustration of the UCNP-based drug delivery system. (**a**) As-synthesized oleic acid capped UCNPs; (**b**) C18PMH-PEG-FA functionalized UCNPs; (**c**) DOX loading on UCNPs. DOX molecules are physically adsorbed into the oleic acid layer on the nanoparticle surface by hydrophobic interactions; (**d**) Release of DOX from UCNPs triggered by decreasing pH. Reproduced from [110]. Copyright 2011, Elsevier Ltd.

Through amphiphilic coating, the multi-functionalization of upconversion nanoparticles could also be realized. For instance, Feng and co-workers fabricated upconversion detection nanocomposites, which were formed by coating the amphiphilic polymer (C18PMH-PEG) on the NaYF_4_:Yb/Er,Tm nanophosphors based on the hydrophobic–hydrophobic interaction. The polymer modified nanoparticles were then assembled for the selective luminescence detection of mercury ions in water. Using the ratiometric upconversion luminescence emission as a detection signal, the detection limit of Hg^2+^ for this nanoprobe in aqueous solution is 8.2 ppb, which is much lower than that (329 ppb) determined by the UV/Vis technology [109]. Liu modified UCNPs with a polyethylene glycol (PEG) grafted amphiphilic polymer, via a hydrophobic interaction-based supramolecular chemistry strategy, for targeted intracellular drug delivery and UCL imaging. This work reveals the great potential of UCNPs for multifunctional drug delivery and biomedical imaging applications (Figure 9) [110]. In contrast to the conventional methods, K. Prud’homme group [111] reported the successful preparation of colloidal UCNPs stable in buffers and serum media (Leibovitz L-15 media with added fetal bovine serum) using FNP and PEG surface coatings. These polymer-modified UCNPs provide promising new materials for applications in bioimaging and photodynamic therapy.

The polymer-coating method is easier when compared to SiO_2_ encapsulation. The *in-situ* coating of polymers on the surface of UCNPs sheds light on the development of multi-functional upconversion nanoparticles. However, the coating through hydrophobic interaction is not stable, and better strategies for a robust polymer coating are needed to satisfy various application requirements.

### 3.3. Ligand Oxidation

**Figure 10:**
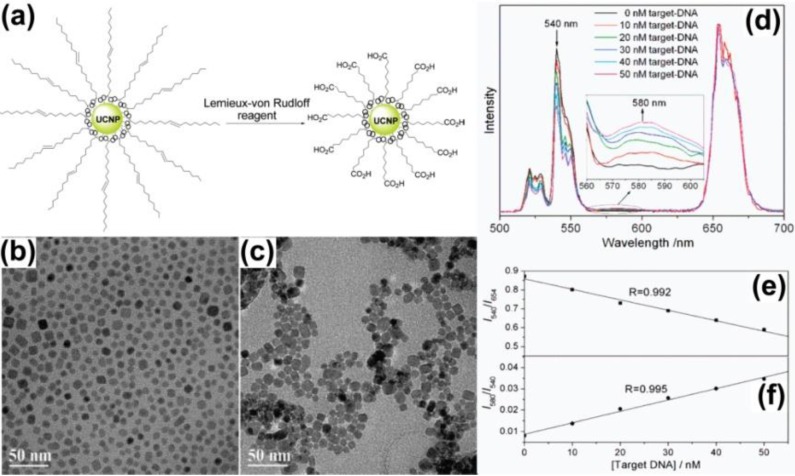
(**a**) Schematic illustration of the ligand oxidization process; (**b,c**) TEM images of the NaYF_4_:Yb/Er nanoparticles before and after ligand oxidization, respectively; (**d**) Luminescence spectra of a mixture of streptavidin-functionalized NaYF_4_:Yb/Er nanoparticles, capture-DNA, and reporter-DNA in the presence of different concentrations of target-DNA under continuous-wave excitation at 980 nm. The linear relationships between target-DNA concentration and the intensity ratios of (**e**) I540/I654 and (**f**) I580/I540. Reproduced from [112]. Copyright 2008, American Chemical Society.

The ligand oxidation technique involves oxidation of the native ligands that contain unsaturated carbon-carbon bonds to generate a pendant hydrophilic functional group. It is reported that a large number of carboxylic acid groups can be generated on the UCNPs surface after oxidation, which not only renders the nanocrystals good solubility in water, but also provides tailorability to biological molecules via direct chemical coupling (Figure 10). It is also reported that the oxidation process has no obvious negative effect on the shape, crystal structure, chemical composition, and luminescence properties of the upconversion nanomaterials [112]. However, this method is limited only to those ligands that contain unsaturated carbon-carbon bonds. The oxidation process may also cause ligand removal due to the harsh experimental conditions. It would be ideal if the modulation of ligand could be achieved under mild experimental conditions, which cause no or less damage to the surfaces of the UCNPs. Biomedical research faces a major challenge in that the lengthy period of oxidation may lead to the excessive formation of the brown MnO_2_ side product, which is not easy to separate and further weakens the upconversion fluorescence [23]. So, the surface functionalization of UCNPs to render these nanoparticles dispersible in aqueous media is very important. Capobianco and co-workers show that the Ln-OA (Ln = lanthanide; OA = oleate) surface of the Ln-UCNPs is replaced by Ln-OH whose state of charge can be tuned by pH and the efficiency of upconversion luminescence can be enhanced by replacing OH with OD. Furthermore, they have studied the effect of different acids, *i.e.*, HCl, H_3_PO_4_, and HF on the surface properties of the oleate-free Ln-UCNPs [113].

### 3.4. Ligand Exchange

**Figure 11:**
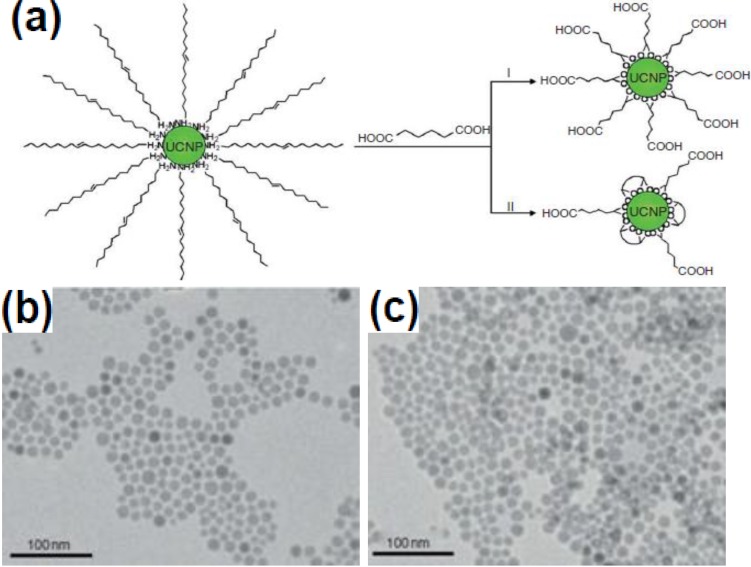
(**a**) Schematic illustration showing the ligand-exchange reactions on OM-stabilized upconversion nanophosphors. (**b,c**) TEM images of NaYF_4_:Yb/Er nanophosphors prior to and after ligand exchange reactions, respectively. Reproduced from [114]. Copyright 2006, John Wiley and Sons.

Ligand exchange involves the displacement of original hydrophobic ligands that have weak coordination with the RE ions on the surface of UCNPs, by ligands that have stronger coordination capability (with the RE ions) and hydrophilic functional groups. As an early example, Chow and co-workers [114] have demonstrated the preparation of water-soluble NaYF_4_:Yb/Er nanoparticles with oleylamine ligands via the ligand exchange method (Figure 11). A large variety of ligands have been reported, including poly(acrylicacid) (PAA) [115], poly(ethyleneglycol) (PEG)-phosphate [116], mercaptopropionic acid (MPA) [117], citrate [118]. Li’s research group [119] demonstrated a new generation of ^18^F-labeled lanthanide nanoparticles of NaYF_4_ co-doped with Gd^3+^/Yb^3+^/Er^3+^ as multimodality nanoprobes for PET, MR and UCL imaging. The presence of Yb^3+^ and Er^3+^ co-doped in the NaYF_4_ nanoparticles gives rise to intense UCL emission in the visible region for luminescent imaging, and 60% Gd^3+^ doping provides the paramagnetic relaxation for MRI. Successful labeling of the lanthanide nanoparticles with ^18^F gave a product suitable for PET imaging [119]. It is noted that after the ligand exchange, most of these commonly used ligand molecules on the UCNP surface carry additional functional groups to facilitate further biofunctionalization and bioconjugation. However, the exchange efficiency of the technique is difficult to evaluate since the surfactant molecules cannot be completely replaced by the oleylamine molecules, and the exchange process is also affected by the pH value of the solution, the concentrations of both the surfactant and the oleylamine molecules, and even the ionic strength.

Besides the above surface modification strategies, Prabhas V. Moghe and coworkers [120] have developed a new approach to rendering NaYF_4_:Yb,Er nanoparticles stable in coacervated HSA nanoshells functionalized with cyclic arginine-glycine-aspartic acid (cRGD) tripeptide. They observed that the composite particles were highly biocompatible *in vitro*, capable of selectively targeting cancerous cell lines exhibiting higher expression of cancer-specific integrin markers, and amenable to fluorescence imaging with high fidelity.

## 4. Conclusions

In summary, this review has summarized the recent development of UCNPs with emphasis on the synthetic methods and the surface modification strategies. With careful control of the reaction conditions, we can now obtain high quality, monodispersed UCNPs with various chemical components. However, each individual synthetic strategy has their unique advantages associated with substantial shortcomings. Our subsequent goal is to develop a general synthetic approach which could meet the requirements of large scale synthesis and multifunctionalities for UCNPs.

In parallel, the controlled surface modification method now can produce UCNPs with high colloidal stability, biocompatibility, and tailorable chemical functionalities. The above discussion has also demonstrated that the rapid development of surface modification method facilitates the applications of UCNPs in detection, bioimaging, therapy, and solar cells. Despite the rapid development, these exiting methods still can only be used in a narrow scope. Strategies with universal comparability for various UCNPs may be the next goal of this field. Alternatively, methods that provide easy surface modulation capability for certain types of nanocrystals could be another option for multifunctionalization.
